# A Newborn Screening, Presymptomatically Identified Infant With Late-Onset Pompe Disease: Case Report, Parental Experience, and Recommendations

**DOI:** 10.3390/ijns6010022

**Published:** 2020-03-14

**Authors:** Raymond Y. Wang

**Affiliations:** 1 Division of Metabolic Disorders, CHOC Children’s Specialists, Orange, CA 92868, USA; rawang@choc.org; 2 Department of Pediatrics, University of California-Irvine School of Medicine, Orange, CA 92868, USA

**Keywords:** Pompe disease, late-onset, infantile-onset, newborn screening, presymptomatic, c.-32-13T&gt, G

## Abstract

Pompe disease is an inherited lysosomal storage disorder caused by acid alpha-glucosidase (GAA) enzyme deficiency, resulting in muscle and neuron intralysosomal glycogen storage. Clinical symptoms vary from the severe, infantile-onset form with hypertrophic cardiomyopathy, gross motor delay, and early death from respiratory insufficiency; to a late-onset form with variable onset of proximal muscle weakness and progressive respiratory insufficiency. Newborn screening programs have been instituted to presymptomatically identify neonates with infantile-onset Pompe disease for early initiation of treatment. However, infants with late-onset Pompe disease are also identified, leaving families and physicians in a state of uncertainty regarding prognosis, necessity, and timing of treatment initiation. This report presents a 31 5/7 weeks’ gestational age premature infant flagged positive for Pompe disease with low dried blood spot GAA activity; sequencing identified biparental c.-32-13T>G/c.29delA *GAA* variants predicting late-onset Pompe disease. The infant’s parents’ initial reactions to the positive newborn screen, subsequent experience during confirmatory testing, and post-confirmation reflections are also reported. While uncertainties regarding natural history and prognosis of presymptomatically-identified late-onset Pompe disease infants will be elucidated with additional experience, suggestions for education of first-line providers are provided to accurately communicate results and compassionately counsel families regarding anxiety-provoking positive newborn screen results.

## 1. Introduction

Pompe disease, caused by acid α-glucosidase enzyme deficiency (GAA; EC 3.2.1.20) due to pathogenic variants in *GAA*, is characterized by intralysosomal accumulation of glycogen throughout bodily tissues, most notably within cardiac and skeletal muscle.

Muscle lysosomal glycogen storage results in hypertrophic cardiomyopathy and skeletal muscle weakness varying in age of onset and severity according to residual GAA enzymatic activity. Skeletal myopathy is also augmented by spinal cord anterior horn and brain neuronal glycogen storage and dysfunction [1]. Infantile-onset Pompe disease (IOPD), caused by near-absence of lysosomal GAA enzyme, typically manifests in the first two months of life with progressive and severe hypertrophic cardiomyopathy, heart failure, marked hypotonia, respiratory failure; if untreated, death from cardiopulmonary complications typically occurs within the first 14 months of life [2].

Late-onset Pompe disease (LOPD) patients have pathogenic *GAA* variants that reduce, but do not completely abolish, acid α-glucosidase enzyme activity. The residual enzyme attenuates the disease course; prior to initiation of newborn screening programs for Pompe disease, LOPD patients were often diagnosed in adulthood following decades of proximal myopathic symptoms. Without treatment, LOPD patients experience deterioration of skeletal muscle strength, becoming nonambulatory and dependent upon artificial ventilation (non-invasive positive airway pressure, or invasive mechanical ventilation) [3].

Pompe disease patients typically have elevations of serum creatine phosphokinase and transaminases secondary to myopathic injury. However, these tests are neither sensitive nor specific for Pompe disease, nor is urinary hexose tetrasaccharide, which is an excreted marker of glycogen accumulation. A diagnosis of Pompe disease relies upon identification of deficient white blood cell GAA enzymatic activity and biparentally inherited variants in *GAA* [4].

Development of intravenous enzyme replacement therapy (ERT) in the form of recombinant human GAA (rhGAA) enzyme, has radically transformed the natural history of Pompe disease. The efficacy of rhGAA ERT is well documented for both IOPD and LOPD. IOPD patients’ overall survival and ventilation-free survival are drastically prolonged; early initiation of rhGAA therapy is associated with better developmental and survival outcomes [5]. Since immune response and cross-reactive immunologic material (CRIM) status play a significant role in outcomes, pre-rhGAA immunomodulation is also indicated to mitigate the neutralizing effect of the anti-rhGAA immune response [6]. LOPD patients also demonstrate improvement in ambulatory and pulmonary function, with delayed onset of declines in six-minute walk and pulmonary function testing [7].

Given the significant improvement in survival, hypertrophic cardiomyopathy, and pulmonary outcomes in infantile-onset patients, Pompe disease was included in newborn screening (NBS) programs around the world. Taiwan, which has been screening for Pompe disease since 2005 due to a founder effect for IOPD [8], reported 28 newborns affected with either form of Pompe disease out of 473,738 screened (1 case per 16,919 newborns) [9]. Surprisingly, prevalences from NBS programs without known Pompe disease founder effects have also been much higher than expected. The Austrian program identified 4 Pompe disease infants out of 34,736 screened (1 case per 8,684 newborns) [10]. Pilot NBS programs in the United States also identified a high prevalence of Pompe disease; the state of Missouri identified 8 affected out of 43,701 screened (1 case per 5463 newborns) [11], while the state of Illinois identified 10 affected out of 219,973 screened (1 case per 21,979 newborns) [12].

Favorable outcomes of NBS-identified IOPD infants in these programs led to the eventual inclusion of Pompe disease to the United States Health and Human Services “Recommended Uniform Screening Panel” in 2013, leading to the implementation of universal newborn screening for Pompe disease in the United States. NBS for Pompe disease began in the state of California in July of 2018.

New challenges emerge with the implementation of every newborn screening program. Quantification of GAA enzymatic activity in NBS samples cannot distinguish between IOPD and LOPD patients. Consequently, for each IOPD case identified, there are anywhere from 1 to 4 LOPD cases identified. If GAA enzymatic activity alone is utilized as the first-tier analyte, heterozygous and pseudodeficiency-carrying infants are also identified. Though rapid initiation of rhGAA ERT is clearly beneficial for IOPD babies, the management—including potential decisions regarding ERT—of NBS-identified, presymptomatic LOPD infants is not clearly defined [13].

Because NBS for Pompe disease was recently introduced in the United States, a clearer understanding of early childhood LOPD phenotypes and natural history will require more time for additional cases to be identified and followed longitudinally. Only then can clear guidelines for clinical follow-up and potential initiation of ERT be issued.

These early uncertainties present difficulties for the clinicians who are the initial evaluators of NBS-identified LOPD infants, and create anxiety and distress for the infants’ parents. This scenario is not novel to the practice of newborn screening: Following implementation of universal screening for phenylketonuria, another inborn error of metabolism for which early institution of treatment significantly impacts outcome, parents of infants ultimately found to be false positives for the disorder demonstrated long-lasting anxiety about their baby’s health [14]. In the era of tandem mass spectrometry-based NBS for multiple inborn errors of metabolism, infants with NBS false positives were hospitalized twice as often as infants with normal NBS [15] due to higher parental stress levels, dysfunction, and perceptions of fragility. If such a possibility for psychological harm exists for parents of infants with false positives, it is undoubtedly present for parents of infants with attenuated conditions for which no clear standard of care yet exists.

The purpose of this report is to highlight one case of LOPD identified through the California Newborn Screening program. More importantly, this report aims to gain insight into how the infant’s parents initially responded to the revelation of their child’s condition, and how they processed and coped with the realization that their child had LOPD. Their goal in sharing their experience and suggestions, which have been curated, is to help all medical staff involved in the newborn screening process—not only medical geneticists, but also the primary care pediatricians’ offices and neonatal intensive care units—better understand the complexities of screening for a heterogeneous disorder and how to effectively counsel and communicate with families whose infants have been identified by NBS.

## 2. Case Report

Parents of the proposita have provided consent for publication of clinical data. The proposita is a second twin, born at 31 5/7 weeks’ gestational age with a birth weight of 1550 g. Her mother was a 36-year-old gravida 2/para 1-2-0-3 woman whose twin pregnancy was monitored closely due to anti-protein S antibodies; she experienced prolonged premature rupture of membranes leading to the proposita’s delivery via repeat Cesarean section. Prenatal screening included normal prenatal nuchal translucency, first trimester laboratory testing, and monitoring ultrasounds.

Initial Apgar scores were 7 at 1 min and 8 at 5 min. She experienced an uncomplicated neonatal course; in the perinatal period, she received empiric antibiotics, phototherapy, and was placed on nasal continuous positive airway pressure. She did not require intubation or supplemental oxygen, and was hospitalized for a total of four weeks in the neonatal intensive care unit advancing oral feedings.

Her newborn screening was flagged for potential Pompe disease as blood spot GAA enzymatic activity was low at 2.7 nmol/mg protein/h (reference range 20.9–140.7). Control β-galactosidase enzyme was within normal range. Blood creatine phosphokinase level was 134 units/liter (reference range <235 units/liter). While urinary hexose tetrasaccharide (uHex4) and *GAA* molecular sequencing were pending, an echocardiogram was performed which documented normal atrial and ventricular dimensions, normal left ventricular wall thickness without evidence of cardiomyopathy, and a normal left ventricular ejection fraction of 72.4%.

Initial uHex4 level was elevated at 25.2 mmol/mol creatinine (reference range <17.6 mmol/mol creatinine); because this was obtained when she was 32 5/7 weeks’ adjusted gestational age, when low urinary creatinine excretion of a premature infant could interfere with accurate measurement, it was repeated five weeks later which was normal at 4.1 mmol/mol creatinine. *GAA* sequencing identified two pathogenic variants: the paternally inherited “common” late-infantile onset c.-32-13T>G variant, and the maternally inherited c.29delA (p.His10Profs*33) variant.

Subsequent measurements of creatine phosphokinase continued to be normal at 215 units/L (23 days of life) and 212 units/L (2.5 months of life). An echocardiogram performed at two months of life continued to show normal ventricular dimensions and function without left ventricular hypertrophy. She had retinopathy of prematurity, which subsequently resolved. She also had apnea of prematurity, for which she was initially placed on caffeine citrate while hospitalized, and subsequently monitored with an apnea monitor after two episodes of apnea following discharge.

Prior to her first evaluation with the author, she was rehospitalized twice for respiratory issues. The first, occurring at 6 weeks of age, was due to respiratory distress caused by rhinovirus bronchiolitis, for which she was placed on nasal continuous positive airway pressure with supplemental oxygen. The second, occurring at 8 weeks of age, took place because she experienced apnea at home requiring cardiopulmonary resuscitation. She was hospitalized for intravenous antibiotics for treatment of presumptive pneumonia and for observation.

Following her hospitalizations, she experienced good subsequent weight gain without any feeding difficulties. At approximately four months of age (two months adjusted for prematurity), her weight was 5.13 kg (50th percentile), length was 54.6 cm (15th percentile), and head circumference 39.5 cm (75th percentile). Examination identified head lag, absence of calf pseudohypertrophy, normal patellar/biceps/triceps/brachioradialis deep tendon reflexes, and reduced truncal and appendicular muscle tone.

## 3. Parental Perspective

We had finally escaped the fluorescent lighting and beeps of the machines in the NICU for a brief moment to take our older son and my mother out for a well-needed frozen yogurt. The phone rang—with the number of the hospital on the screen—sending chills down my spine. The doctor informed us that one of our twins, who were born two months prematurely, had an abnormality on the newborn screening test. “What disease and what baby?” I asked.

“Pompeii disease in baby B” he seemed to have said. Images of an entire civilization frozen in molten lava came flooding through my mind. “It’s a metabolic disease. It’s most likely a false positive. We see this a lot. Once the baby eats and the metabolism stabilizes, the test will most likely be normal. We will confirm with a genetic test and a few other tests. Just don’t look it up online.” So, of course, immediately, my husband and I looked it up. Autocorrect changed “Pompeii” to “Pompe.” From the (now I understand outdated) catastrophic and deadly description of the disease online, I would have preferred a Roman volcanic eruption.

We would later learn that this description was for IOPD before the current enzyme replacement therapy treatments, which have dramatically improved the outcome for Pompe disease patients. We would also later learn that there are at least two very distinct types of Pompe disease, infantile, and late-onset, and that doctors, researchers, and biotech companies have made tremendous strides in understanding, treating, and halting the progression of the disease. Some born with IOPD can walk, dance, sing, and even attend college. Late-onset patients are now identified more quickly due to newborn screening, and can be treated before muscular deterioration occurs. I have since met a successful scientist, a neurologist, and the head of a foundation who all have LOPD, and you would most likely never know they had it. Furthermore, with gene therapy clinical trials already accepting applicants and gene editing on the horizon, there is even the prospect of the eliminating the disease entirely.

However, at this point, we were only seeing what was online. “It’s a lysosomal storage disease”, I said to my mother while driving back in the car. She stopped the car, paralyzed. “That’s what your brother had. It’s a different gene but the same category of disease.” How could this be? My brother had died of Batten Disease, and my husband and I underwent extensive genetic counseling to help insure that none of our children would have a rare genetic disease.

What I found out later about prenatal carrier testing was that I was only tested for the common variant of Pompe disease (c.-32-13T>G), which I do not carry, but my husband does. I later discovered that I carry a previously unrecorded, but most likely very severe, mutation that contains a deletion. In our prenatal counseling, my husband was not tested as a carrier for Pompe disease at all. One counselor had told me I had the same risk as the general population. Another doctor said it would be unlikely that I would carry Pompe disease because I was not of Dutch descent.

When we did receive the genetic confirmation that our baby did indeed have the disease, the genotype–phenotype correlation baffled the NICU doctors and the consulting geneticist. She had the common variant (predicting late-onset Pompe disease) and this unrecorded severe mutation (predicting infantile Pompe disease). Whereas classic IOPD, defined by cardiomyopathy as well as low GAA enzymatic levels in an infant younger than one year of age, is very severe, LOPD can be potentially mild with a later multi-symptom disease progression. Cardiac involvement is rarely involved in the c.-32-13T>G mutation [16]. In one study, ERT was initiated early with some LOPD infants exhibiting very mild early signs of muscle weakness [17].

However, because one of our child’s confirmatory tests were abnormal with elevated uHex4 levels, which could indicate glycogen storage, the doctors did not know whether the baby had infantile or late-onset Pompe disease. Although infantile Pompe disease is sometimes known as “floppy baby syndrome,” she was not floppy at all but had excellent muscle tone. Her treatment depended upon a correct diagnosis: infantile-onset Pompe disease needed enzyme replacement therapy treatment right away, while for late-onset Pompe disease, enzyme replacement therapy was less urgent. The laboratory performing the uHex4 test informed us that this elevation was due to prematurity; thankfully, in the next test, the uHex4 levels were normal.

There was quite a learning curve with terms like “uHex4,” “ERT (enzyme replacement therapy)”, “leaky splice site,” “c.-32-13T>G,” “GAA-levels”, “CK levels,” “exon,” “del (deletion),” and “dup (duplication)”, but after a few months of research, we found ourselves able to understand much of the genetic lingo and code. I found it most helpful to start with the articles referencing the specific alleles (if previously reported), which were cited in the genetic confirmation of the disease. This evolved to understanding broader issues and concepts in the Pompe community by attending conferences focusing on Pompe or rare disease and by making contact with leading researchers, doctors, and biotech companies interested in Pompe as well as parents of kids with Pompe and patients with Pompe.

We also researched observational studies and clinical trials. There are many groups on social media to connect people as well. In the rare disease community, we discovered, as one patient put it, “patients and parents are treated as collaborators.” There is so much hope in this field, and no parent needs to be devastated by the newborn screening results. In fact, we are also filled with gratitude that this disease was caught early and can be treated and eventually eliminated.

## 4. Suggestions from Parents, to Medical Staff Counseling or Seeing Families Screening Positive for Pompe NBS

### 4.1. Screening and Genetic Counseling

Do not minimize the initial results for the parents by telling them that chances are the test is a false positive. The follow up testing may in fact demonstrate that the initial result was in fact a true positive.

Do not tell parents that infantile Pompe disease means that the child will die as an infant.

Do not tell parents not to look up the disease online. Instead, give them suggestions of what to look up and what to ignore.

A person does not have to be Dutch to carry a Pompe disease gene mutation.

A baby that “looks healthy” can still have a genetic disease.

The common c.-32-13T>G variant of Pompe, even when present on one allele with a normal echo, does not result in IOPD. It predicts later onset Pompe disease.

### 4.2. Pompe Disease Management and Treatment

Do not early discharge premature infants from the NICU if Pompe disease, or any other serious metabolic disorder, is suspected. The geneticist should perform a clinical evaluation and examination of the child. Prematurity should also be closely monitored.

Teach other health care staff about the difference between infantile and late-onset Pompe disease.

Learn about enzyme replacement therapy and inform the parents about it.

IOPD babies must be treated with enzyme replacement therapy as soon as possible once CRIM status has been confirmed and immunomodulation potentially initiated [6].

Give a referral to a dietician versed in Pompe disease. Diets should be high in protein but low in simple carbohydrates [18].

Give a referral to physical therapy if possible and if necessary. We have found that physical therapy can help build up muscle strength even without ERT yet.

### 4.3. Family Advocacy

Tell the parents about all the incredible resources there are for Pompe patients, which will inform parents of gene therapy studies, observational newborn studies, clinical trials, next generation ERT therapies, pharmacological chaperones and put them in touch with the Pompe community.

Tell parents to research and contact the leading Pompe doctors, researchers, and biotech companies in the country.

Inform the parents about registries.

Tell the parents to have hope! Pompe disease has been thoroughly researched. It is precisely because there is treatment that it is on the NBS, and the one word that continuously circulates in discussions of upcoming and eventual gene therapies for Pompe disease is “cure.”

## 5. Discussion

This report highlights a case of a neonate in the State of California screening positive for Pompe disease. Reported herein are findings of clinical, biochemical, and molecular confirmatory testing that ultimately identified a diagnosis of late-onset Pompe disease. Additionally reported are the reactions and emotional journey taken by the infant’s parents from initial notification, to definitive diagnosis, and subsequent follow-up.

The child’s neonatal course was complicated by prematurity, not only due to premature physiology resulting in increased excretion of urinary hexose tetrasaccharide, but also due to apnea of prematurity episodes/intercurrent illness in which she required supplemental oxygen or cardiopulmonary resuscitation. She has hypotonia on physical examination, and it is too early to tell if the hypotonia is a result of her prematurity, potential neurologic sequelae from hypoxia, or due to early symptoms of LOPD.

Review of clinically-diagnosed (not NBS-identified) Pompe disease patients indicates that patients with the common c.-32-13T>G intronic splice site *GAA* variant uniformly develop LOPD are very rarely develop childhood-onset cardiac disease [16]. While most of these patients develop myopathic symptoms and are at risk of dysrhythmias and left ventricular hypertrophy in adulthood, a small subset may manifest gross motor delay and proximal muscle weakness during their first years of life. A retrospective review of 84 symptomatically-identified LOPD patients with at least one c.-32-13T>G variant identified four patients presenting with symptoms prior to 20 months of age; of these patients, three had elevated creatine phosphokinase and two had elevated uHex4 levels [19]. An examination of 7 NBS-identified LOPD patients with the c.-32-13T>G variant demonstrated that while all infants had some degree of muscle weakness, the four patients with the most notable weakness had elevations in creatine phosphokinase. All NBS-identified infants had normal uHex4 levels [17].

For the infant in this report, physical therapy was recommended due to her hypotonia, prematurity, apnea of prematurity, and LOPD diagnosis. Quarterly monitoring for signs and symptoms of spinal and pelvic girdle muscle weakness, calf pseudohypertrophy, as well as laboratory quantification of transaminases, creatine phosphokinase, and uHex4 was instituted. Given the difficulties of secure intravenous access, potential for infusion-associated reactions, and absence of creatine phosphokinase/uHex4 elevation at assessment, ERT was deferred. The infant is now considered a “patient-in-waiting”, with her parents and her metabolic physician placing her under surveillance for development of symptoms, “living between health and disease” and unable to determine when treatment should be instituted [20].

Pompe disease was added to newborn screening panels in order to rapidly identify and initiate treatment for neonates with the severe, infantile-onset form of the disease. Cardiac, motor, and overall survival outcomes in early-treated IOPD babies is superior to outcomes in IOPD babies initiated with ERT after symptomatic presentation [13]. However, since NBS identifies neonates with LOPD as well as those bearing pseudodeficiency variants and *GAA* variant heterozygotes, the implementation of NBS for Pompe disease has created considerable uncertainty. First, for parents of NBS-flagged infants, who must wait for results of confirmatory testing and undoubtedly experience some degree of anxiety after reading about the morbidity and mortality associated with Pompe disease; second, for medical providers—especially primary care practitioners and non-physician staff—who are often tasked with notifying parents about NBS results, but may feel their knowledge about Pompe disease is inadequate and subsequently either minimize the significance of the NBS or transmit their apprehension to the parents. Finally, after confirmation of LOPD, uncertainty exists for parents and specialty caregivers as there is currently no established management guideline for presymptomatically-identified LOPD infants. Our family’s experience mirrors closely those of other families receiving Pompe disease NBS results [21]. As additional LOPD cases are identified and followed longitudinally with existing NBS-identified LOPD cases, a clearer natural history, prognosis, and recommendations for timing of treatment initiation will arise. Increased knowledge will help allay some of the anxieties and uncertainties faced by families of NBS-identified LOPD babies. 

It is our parents’ hope that their suggestions, which we are in agreement with, and the experience of other families will enhance health care practitioners’ abilities to accurately communicate results and compassionately counsel families regarding anxiety-provoking positive newborn screen results.
